# Effect of Whole Tissue Culture and Basic Fibroblast Growth Factor on Maintenance of Tie2 Molecule Expression in Human Nucleus Pulposus Cells

**DOI:** 10.3390/ijms22094723

**Published:** 2021-04-29

**Authors:** Kosuke Sako, Daisuke Sakai, Yoshihiko Nakamura, Jordy Schol, Erika Matsushita, Takayuki Warita, Natsumi Horikita, Masato Sato, Masahiko Watanabe

**Affiliations:** 1 Department of Orthopedic Surgery, Tokai University School of Medicine, 143 Shimokasuya, Isehara, Kanagawa 259-1193, Japan; sato-m@is.icc.u-tokai.ac.jp (M.S.); masahiko@is.icc.u-tokai.ac.jp (M.W.); 2 Center for Musculoskeletal Innovative Research and Advancement (C-MiRA), Tokai University Graduate School, 143 Shimokasuya, Isehara, Kanagawa 259-1193, Japan; 3 Research Center for Regenerative Medicine, Tokai University School of Medicine, 143 Shimokasuya, Isehara, Kanagawa 259-1193, Japan; kahiko@is.icc.u-tokai.ac.jp (Y.N.); schol.j@tsc.u-tokai.ac.jp (J.S.); silsilsilring@gmail.com (E.M.); takayuki.warita@tunzpharma.co.jp (T.W.); natsumi.horikita@tunzpharma.co.jp (N.H.)

**Keywords:** intervertebral disc, nucleus pulposus, nucleus pulposus progenitor cell, Tie2 receptor, basic fibroblast growth factor, chimera fibroblast growth factor, ERK, Akt, whole tissue culture, type II collagen

## Abstract

Previous work showed a link between Tie2^+^ nucleus pulposus progenitor cells (NPPC) and disc degeneration. However, NPPC remain difficult to maintain in culture. Here, we report whole tissue culture (WTC) combined with fibroblast growth factor 2 (FGF2) and chimeric FGF (cFGF) supplementation to support and enhance NPPC and Tie2 expression. We also examined the role of PI3K/Akt and MEK/ERK pathways in FGF2 and cFGF-induced Tie2 expression. Young herniating nucleus pulposus tissue was used. We compared WTC and standard primary cell culture, with or without 10 ng/mL FGF2. PI3K/Akt and MEK/ERK signaling pathways were examined through western blotting. Using WTC and primary cell culture, Tie2 positivity rates were 7.0 ± 2.6% and 1.9 ± 0.3% (*p* = 0.004), respectively. Addition of FGF2 in WTC increased Tie2 positivity rates to 14.2 ± 5.4% (*p* = 0.01). FGF2-stimulated expression of Tie2 was reduced 3-fold with the addition of the MEK inhibitor PD98059 (*p* = 0.01). However, the addition of 1 μM Akt inhibitor, 124015-1MGCN, only reduced small Tie2 expression (*p* = 0.42). cFGF similarly increased the Tie2 expression, but did not result in significant phosphorylation in both the MEK/ERK and PI3K/Akt pathways. WTC with FGF2 addition significantly increased Tie2 maintenance of human NPPC. Moreover, FGF2 supports Tie2 expression via MEK/ERK and PI3K/Akt signals. These findings offer promising tools and insights for the development of NPPC-based therapeutics.

## 1. Introduction

In our aging societies, low back pain has developed as a primary cause of disability and often leads to poor quality of life for patients [1,2]. Low back pain is thought to relate to intervertebral disc (IVD) degeneration, although the mechanisms have not been clearly identified [3]. The IVD is composed of three distinct tissues: the nucleus pulposus (NP), the annulus fibrosus (AF), and cartilage endplates. The NP forms the gelatinous tissue located in the center of the IVD. The AF comprises layered collagen structures that surround the NP [4]. The AF is characterized by a well-organized network of concentric collagen lamellae, with type I collagen being the predominant extracellular matrix (ECM) constituent [5]. The NP mostly comprises type II collagen and proteoglycans, especially aggrecan, which maintains tissue hydration due to its chondroitin and keratan sulfate chains [6]. The constriction of the highly osmolar NP by the AF gives the IVD its biomechanical function.

The NP- and AF-ECM are continuously replenished and remodeled by tissue-specialized cells and are crucial in supporting the IVD function. However, due to aging, trauma, and genetic and lifestyle factors, the cells can lose their potency and viability, thereby limiting their collective ECM production capacity [3]. The compromised ECM impacts biomechanical features, engendering a further strained and catabolic environment, thereby progressively degenerating the IVD. The catabolic environment causes cells to secrete a range of inflammatory cytokines [3,7,8], which in turn can trigger blood vessel and neuron ingrowth into the previously avascular and un-innervated discs [9,10,11]. This degeneration cascade can subsequently lead to nociception or stenosis and can cause debilitating pain and disability for some patients.

Contemporary treatments remain unable to resolve IVD degeneration. However, a range of upcoming regenerative therapies has been proposed including gene therapy [12], growth factor injection [13], biomaterial injection [14], and tissue engineering [15,16]. As one of the features of disc degeneration is a decline in cell numbers and potency, multiple investigations have examined the potential of introducing active cells into the IVD to re-establish ECM production or temper the catabolic state [17,18]. Multiple clinical trials have confirmed the potential of cell transplantation to alleviate pain, although their regenerative effects remain to be confirmed [17]. Moreover, the optimal cell type and conditions remain a topic of debate [17,19,20].

We have previously identified angiopoietin-1 receptor (Tie2)-positive human NP progenitor cells (NPPC), which showed a strong decline in parallel with IVD degeneration and aging in human patients [21]. These Tie2^+^ cells were characterized as undifferentiated cells with multipotency and possessing high self-renewal abilities [22,23,24,25]. Therefore, we and others have proposed Tie2^+^ NPPC as a potent cell source for regenerative cell therapies against IVD degeneration [26]. However, their utilization is hindered by low Tie2-expressing cell yields from NP tissue, in particular from commonly available older and degenerated tissue sources [21,25]. Moreover, NP-derived cells rapidly dedifferentiate [27,28] and NPPC show a rapid Tie2 decrease due to cell differentiation as part of standard culture processes [23]. As such, a need exists to optimize or develop new culture methods that enable the maintenance of Tie2-expressing NPPC. A variety of studies have investigated different approaches to improve culture conditions [26,29,30,31,32] or isolation methods [22] to increase the number of Tie2-expressing cells. Bovine-derived NP cell studies have suggested a synergistic beneficial role of fibroblast growth factor 2 (FGF2; also known as basic fibroblast growth factor) and a hypoxic culture environment. However, their effect on human NP cells remains to be determined [29]. In particular, the three-dimensional (3D) microenvironment and mechanotransductional niche [33] appear to be critical in regulating Tie2^+^ NPPC abilities. For example, the Tie2^+^ NPPC was originally discovered by their formation of spheroid colony-forming units (CFUs) within a methylcellulose semi-solid medium [21]. Moreover, Zhang et al. reported a high colony formation rate and Tie2 positivity rate by using an ultra-low attachment T75 flask [30]. Similarly, Guerrero et al. recently found enhanced Tie2^+^ NPPC maintenance by employing a 3D environment of alginate-beads with enhanced pluripotency markers. These studies similarly emphasized the rapid decline of Tie2-expressing cells in a standard monolayer culture.

In this study, we aimed to employ the natural NPPC niche present in NP tissue as a culture environment to enhance and maintain NPPC. We investigated the potential of a whole tissue culture (WTC) method and subsequent optimization through FGF2. We also examined the short-term effects of FGF2 removal or FGF chimera (cFGF; a more chemically stable mutant variant of FGF that combines FGF1 and FGF2 [34]). In a previous report, cFGF has been shown to have an effect equivalent to FGF2 in maintenance of pluripotency marker expression, global gene expression, karyotype, and differentiation potential in both human embryonic stem cells and induced pluripotent stem cells. It also exhibits higher thermal stability and protease resistance than both FGF1 and FGF2 [34,35,36]. As such, we examined their potential to support Tie2^+^ NP cell maintenance following our optimized culture. Moreover, although FGF2 stimulates the proliferation of IVD cells via the activation of the MEK/ERK and PI3K/Akt signaling pathways in bovine NP cells [37], its function and the direct relationship with ECM production performance is not confirmed. Accordingly, we set out to determine the role of the MEK/ERK and PI3K/Akt pathways on FGF2-supported Tie2 expression maintenance, and their impact on type II collagen expression in human NP cells. We hypothesized that FGF2 stimulation, as similarly shown in bovine NP cells, would be able to support Tie2^+^ NP cell maintenance and that this was in part effectuated via the MEK/ERK or PI3K/Akt pathway.

## 2. Results

### 2.1. The Effect of WTC Method and FGF2 on Tie2 Expression Rates

**Figure 1:**
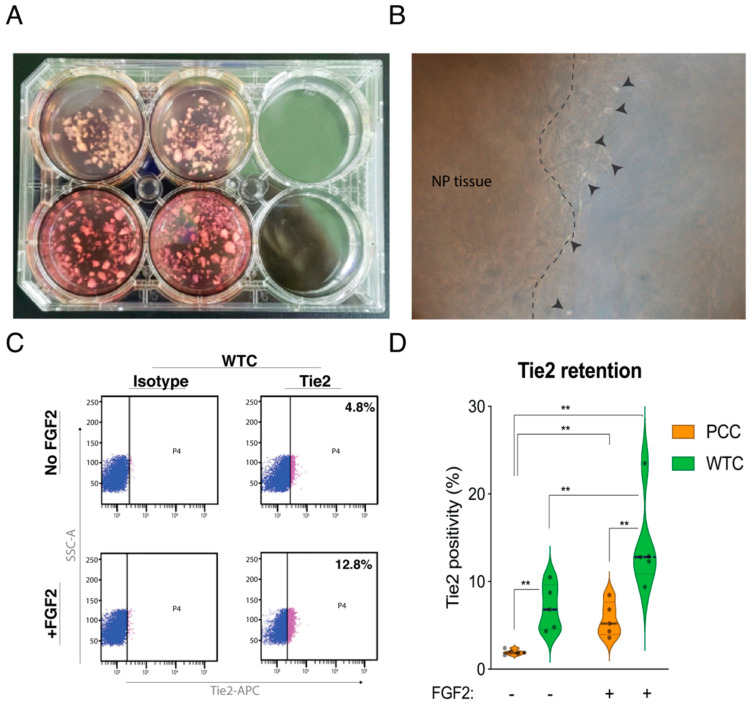
Comparison of primary cell culture (PCC) and whole tissue culture (WTC) methods. (**A**) Human nucleus pulposus (NP) tissue is fragmented into 3–5 mm pieces and suspended in culture medium. (**B**) Following two weeks of culture, cell division and proliferation occurred in tissue without attaching or stretching to the plate. (**C**) Representation of flow cytometry analysis on WTC and PCC NP cells, supplemented with or without FGF2. Gating occurred by on isotype controls [23]. (**D**) Flow cytometry quantitative data (*n* = 5) assessing the fraction of Tie2-expressing NP cell, as median (bar) and quartiles (dotted lines). ** *p* < 0.01.

WTC could successfully be established. Tissue fragments following 14 days of culture remained suspended. Despite not changing the medium, cell populations appeared over time on the margins of the tissue that were devoid of cells at the initiation of the WTC, suggesting cell proliferation (Figure 1B). Flow cytometry analysis (Figure 1C) demonstrated that NP cells obtained following primary cell culture (PCC) and WTC presented on average a Tie2 positivity rate of 1.9% (±0.3%) for PCC and a significantly higher 7.0% (±2.6%) for the WTC method (*p* = 0.004) (Figure 1D). FGF2 supplementation significantly enhanced Tie2 rates in both PCC (5.7% ± 2.0%, *p* = 0.01) and WTC conditions (14.2% ± 5.4%, *p* = 0.01). FGF2-supplemented WTC conditions resulted in a significantly higher Tie2 positivity rate (*p* = 0.004) compared with FGF2 supplemented PCC cultured NP cells (Figure 1D).

### 2.2. Tie2 Expression and Signal Transduction Pathway by cFGF

**Figure 2:**
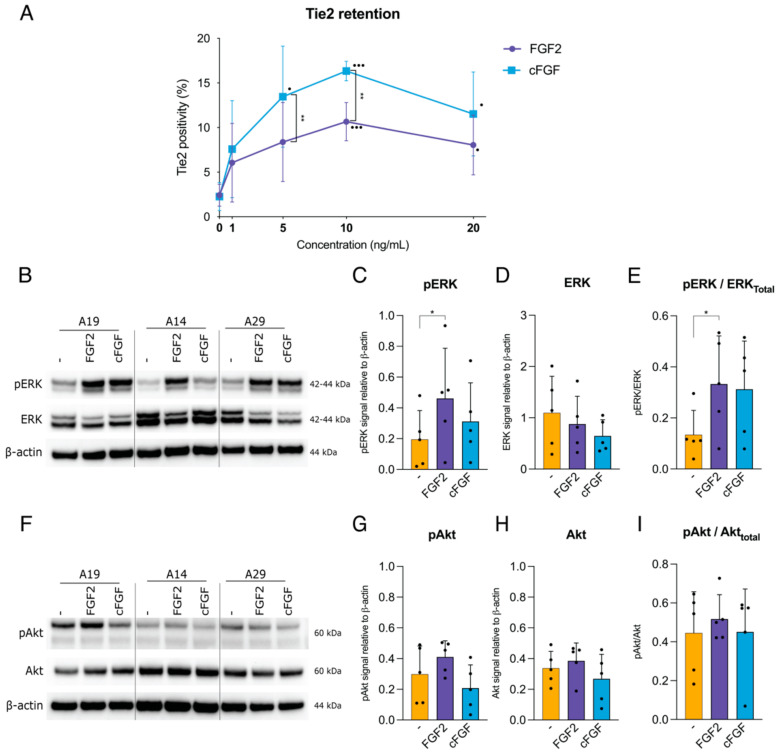
Examination of the involvement of the MEK/ERK and PI3K/Akt pathway in FGF2 and cFGF supported Tie2 retention. (**A**) Flowcytometry (*n* = 5) determined fraction of Tie2 expressing NP cells following FGF2 and cFGF stimulation in non-serum condition for 24 h with a concentration of 1, 5, 10, or 20 ng/mL. ** *p* < 0.01 comparing FGF2 to cFGF, or • *p* < 0.05 ••• *p* < 0.001 compared to 0 ng/mL conditions. (**B**) Representative western blot of three donors (A19, A14, A29, Table 1) for ERK activation, following stimulation with 10 ng/mL FGF2 or cFGF. β-actin was applied as a loading control. Quantification of ERK activation western blot (*n* = 5), presenting (**C**) pERK and (**D**) ERK values relative to β-actin signal. (**E**) Quantification of pERK values relative to total ERK. (**F**) Western blot for three representative donors (A19, A14, A29, Table 1) for Akt activation, following stimulation with 10 ng/mL FGF2 or cFGF. β-actin was applied as a loading control. Quantification of Akt activation western blot (*n* = 5), presenting (**G**) pAkt and (**H**) Akt values relative to β-actin signal. (**I**) Quantification of pAkt values relative to total Akt signal. All values presented as means, and error bars represent standard deviation. * *p* < 0.05—indicates unstimulated cells and • shows each sample data.

Based on the WTC and PCC outcomes, and also taking into account market translatability, we employed standard NP expansion by WTC culture for two weeks without FGF2, and subsequent 2-week monolayer culture with 10 ng/mL FGF2. Following, WTC culture Tie2 expression was determined as on average 10.0% (±3.2). Subsequently, following full expansion, cells were serum starved (to severely limit Tie2 retention), while stimulated with different concentrations of FGF2 and cFGF (a chimeric protein that combines FGF1 and FGF2) to maintain Tie2 expression. Both factors were applied 24 h following expansion and demonstrated an increase in Tie2 expression in a concentration-dependent manner, reaching a plateau at 10 ng/mL (Figure 2A). Tie2 expression was significantly enhanced at 10.7 ± 2.1% with 10 ng/mL FGF2 (*p* = 0.004) and 16.3 ± 1.1% with 10 ng/mL cFGF (*p* = 0.002) compared with non-stimulated NP cells. Comparing FGF2 and cFGF at each concentration, cFGF significantly enhanced Tie2 expression at 5 ng/mL (*p* = 0.007) and 10 ng/mL (*p* = 0.007) concentrations (Figure 2A). Western blotting showed significant ERK phosphorylation in the presence of both FGF2 and cFGF 30 min after addition (Figure 2B). Although inferior to ERK, a faint band was also confirmed for phosphorylated Akt (Figure 2F). Quantification relative to β-actin showed a significant increase in phosphorylated ERK following FGF2 (*p* = 0.03) stimulation compared with the unstimulated control, but cFGF (*p* = 0.123) stimulation did not show significant difference upon statistical analysis (Figure 2C). Relative ERK yields presented no clear differences between conditions (Figure 2D), however, ERK activation (relative to total ERK) was seen for both FGF2 and cFGF conditions, although only FGF2 stimulation resulted in a significant difference (*p* = 0.0434). (Figure 2E). Levels of pAkt and Akt showed some small differences in each condition, but these were not significantly different (Figure 2G,H). Similarly, pAkt levels relative to total Akt showed no enhanced activation by either FGF-type (Figure 2I).

### 2.3. Effects of MEK, Akt Inhibitors on FGF2 Signaling and Tie2 Expression

**Figure 3:**
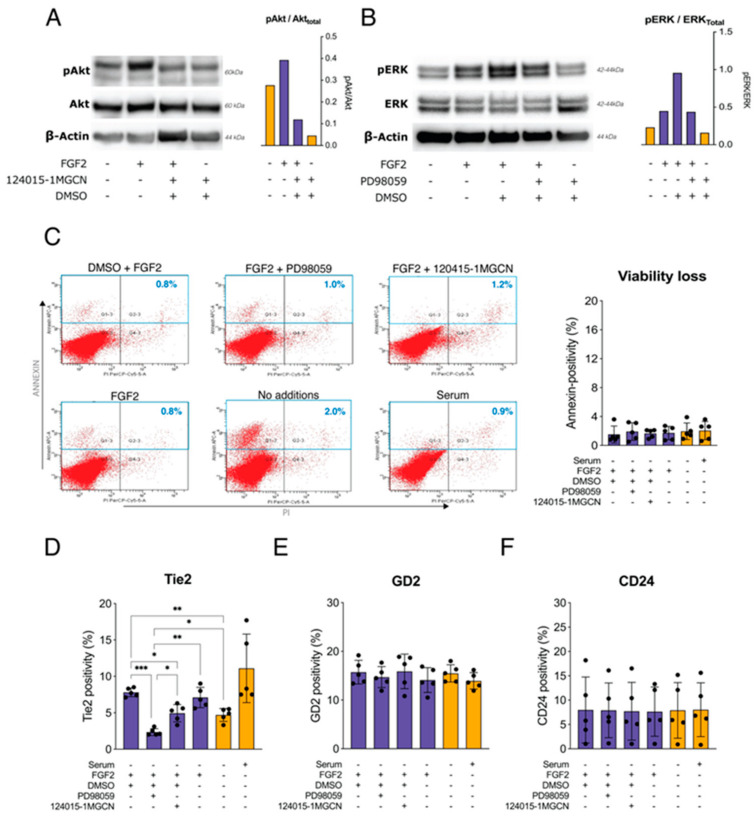
Effect of Akt and MEK inhibition on Tie2 maintenance. (**A**) Western blot to determine the reduced phosphorylation of Akt following FGF2 stimulation (24 h) when subjected to Akt inhibitor 124015-1MGCN (*n* = 1). (**B**) Western blot showing the ability of PD98059, a MEK inhibitor, to interfere with FGF2 induced ERK phosphorylation (*n =* 1). (**C**) Flow cytometry (*n* = 5) analysis based on propidium iodide (PI) and annexin based gaiting to exclude negative effects on viability by 124015-1MGCN and PD98059. Flow cytometry analysis (*n* = 5) determining the fraction of (**D**) Tie2, (**E**) GD2, and (**F**) CD24 expressing NP cells following 24 h of specified culture. Note DMSO was added as a control to compensate for effect potentially caused by the DMSO included in PD98059 and 124015-1MGCN storage solutions. All values presented as means, and error bars represent standard deviation. * *p* < 0.05, ** *p* < 0.01, and *** *p* < 0.005. Purple bars: FGF2 included conditions, Orange bars: FGF2 non-included conditions.

Akt inhibitor 124015-1MGCN could clearly temper FGF2 stimulated phosphorylation of Akt (Figure 3A). By comparing the FGF2 only and FGF2 + DMSO condition, ERK phosphorylation was enhanced by DMSO. Taking these results into account, ERK phosphorylation was shown to be successfully suppressed by the MEK inhibitor PD98059 (Figure 3B).

Next, the impact of the MEK/ERK and PI3K/Akt pathways on FGF2-supported Tie2 expression was examined (Figure 3D). Tie2 positivity rates were 11.1 ± 4.2% without serum-starvation but dropped to 4.7 ± 0.8% when subjected to 24 h serum starvation. Maintaining FGF2 supplementation was able to maintain Tie2-expressing cells at a rate of 7.8 ± 0.5%, significantly (*p* = 0.007) higher than the unstimulated serum-starved cohort. FGF2-stimulated cells treated with PD98059 showed a further decline in Tie2 positivity to 2.3 ± 0.4% (*p* = 0.01, compared with FGF2 alone), while 124015-1MGCN only reduced Tie2 rates to 4.9 ± 1.1% (*p* = 0.42, compared with FGF2 alone). Another NPPC marker, GD2, and an NP cell marker, CD24, showed no evident change in response to any of the treatments (Figure 3E,F). Finally, using annexin and PI-based flow cytometry, the negative impact of the treatments on cell viability was negated (Figure 3C).

### 2.4. Correlation Between Tie2 Expression by FGF2 and COL2A1 Expression

**Figure 4:**
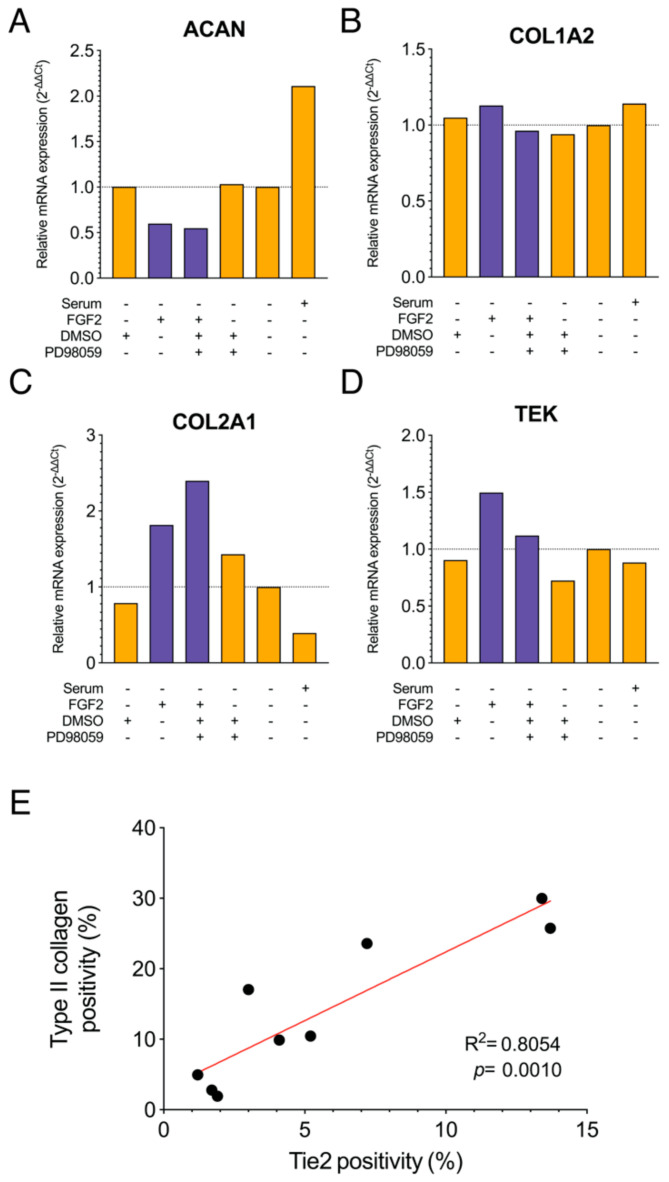
Assessment on the effect of FGF2 on matrix production of potency. RT-PCR data (*n* = 1) for nucleus pulposus (NP) cell (**A**) ACAN, (**B**) COL1A2, (**C**) COL2A1, and (**D**) TEK: gene of Tie2. mRNA expression levels calculated relative to GAPDH, and subsequently to unstimulated serum-starved conditions. Purple bars: FGF2 included, Orange bars: FGF2 non-included. (**E**) Simple linear regression analysis of flow cytometry outcomes correlating fraction of Tie2 positivity and type II collagen positive NP cells.

Regarding the preliminary gene expression assay by real-time PCR, the expression of ACAN (aggrecan gene) tended to decrease with continuation of FGF2 supplementation compared with unstimulated culture conditions (Figure 4A). Expression of COL1A2 did not change markedly under any of the conditions (Figure 4B). The expression of COL2A1 and TEK (Tie2 gene) tended to increase with FGF2 compared with other conditions for which FGF2 stimulation was halted (Figure 4C,D). From this, we decided to verify the possibility that there is a correlation between Tie2 expression and type II collagen production. Nine experiments were performed on three donor samples using flow cytometry. The Tie2 expression rate and the type II collagen expression rate were measured and their correlation was examined. Type II collagen positive cells tended to increase in proportion to the Tie2 expression rate, and a significantly positive correlation was observed between the two (r = 0.8974, R^2^ = 0.8054, *p* = 0.001) (Figure 4E).

## 3. Discussion

In the avascular human NP tissue [9], Tie2^+^ cells characterize a specific undifferentiation and highly potent progenitor cell sub-population [21,23,25,38,39]. Transplanted Tie2^+^ NPPC have been shown to be capable of maintaining subcutaneous acellular NP tissue in a mouse model [21]. NP cells selected based on their CFU ability have similarly been shown to be a highly effective cellular product in a canine disc degeneration model [40]. This cell product is currently being examined in clinical trials in Japan [41] and the USA [42]. The establishment and advancement of culture methods that are able to sustain Tie2^+^ NPPC will thus likely enable optimized regenerative therapeutics. In this study, we employed a classical cell culture method in which we kept the NPPC in their innate niche, supporting NP cell expansion while maintaining a high Tie2 positivity rate. Moreover, when designing culture methods, it is pivotal to consider culture simplicity and cost, both of which are critical hurdles for final therapeutic marketability and their translation to the clinic [17,18,43]. Our culture conditions, which involved two weeks of culture of NP tissue supplemented with or without 10 ng/mL FGF2, strongly enhanced Tie2 expression levels and NP cell potency. For example, FGF2-stimulated WTC culture increased the final Tie2^+^ fraction 7.5-fold compared with standard PCC. Comparatively, work by Guerrero et al. [26] revealed a 1.8-fold increase in Tie2 rates, comparing their human NP cells cultured in alginate bead versus a standard monolayer culture. A 3.6-fold increase was reported by Zhang et al. [30], who employed spheroid-based suspension cultures. Notably, these studies reported different baseline Tie2 rates and culture times. Even so, they highlight that our WTC method offers a promising, cost-effective approach for retaining Tie2 NPPC in culture that is competitive with other explored strategies.

In vascular endothelial cells, the interaction of Tie2 molecules between cells and the involvement of notch signals has been extensively studied [44]. Tie2 and its agonist, angiopoietin (Ang)-1, and antagonist, Ang-2, are recognized for their decisive role in regulating vasculature permeability and endothelial cell maturation [45]. However, their role and regulation in the maintenance of the NPPC phenotype remain elusive. Aside from forming a signaling receptor for Ang-1/Ang-2, Tie2 is also able to form trans-complexes with other Tie2 receptors on neighboring cells, thereby supporting cell adherence and clustering [45]. Although it remains to be validated, it is reasonable to assume that Tie2-mediated cell clustering is a critical aspect for maintaining NPPC. In the original work of Sakai et al. [21], Tie2^+^ NPPC were found clustered together within the IVD. Similarly, contemporary NPPC culture methods [26,30] show the benefits of maintaining NPPC in clusters. The pericellular environment and sequestered factors are presumed to be critical in maintaining Tie2-expressing NPPC; however, this remains a topic for future examination.

In vascular endothelial cells, Akt signaling from FGF2 and that from Tie2 are linked to cell survival and inhibition of apoptosis [46,47]. Similarly, a recent report by Wang et al. demonstrated the role of Ang-2 in IVD degeneration, further confirming that PI3K/Akt signaling plays a critical role in Tie2-regulated NP cell survival [48,49]. More specifically, MEK/ERK and PI3K/Akt pathways have been classified as crucial signaling pathways for rat NP cells to survive under hypoxic conditions [50]. Subsequently, Pratsinis et al. suggested that FGF2 can stimulate NP cell proliferation through the MEK/ERK and PI3K/Akt pathways [37,51,52]. Interestingly, Tekari et al. [29] discovered that FGF2 and hypoxia worked synergistically to enhance Tie2 expression in bovine NP cells. We thus hypothesized a link between FGF2-induced MEK/ERK and PI3K/Akt activation and FGF2-enhanced Tie2 expression. This hypothesis was confirmed by our present study. Our results suggest a clear relation, as FGF2-stimulated NP cells presented an increase in both Tie2 maintenance and an increase in pERK. Moreover, inhibition of MEK through PD98059 limited ERK activation and consequently reduced the rate of Tie2^+^ NP cells. The Akt inhibitor, 124015-1MGCN, also produced a significant decrease in FGF2-supported Tie2 expression, although less pronounced than MEK inhibition. These observations further confirmed our hypothesis that FGF2-mediated NPPC retention was affected via the ERK/MEK and to a lesser extent, the PI3K/Akt pathways. More specifically, throughout our study, we were able to demonstrate that even a 24 h removal of FGF2 from the media was able to severely and significantly impact Tie2 expression. Since both pathways play a crucial role in regulating cell survival, we confirmed that both treatments did not result in a loss of cell viability. PD98059 was observed to have no effect on cell viability, as shown in Figure 3C. Moreover, Akt inhibition at different concentrations of 124015-1MGCN also did not impair cell viability up to a concentration of 1 μM (Figure S1).

Additionally, we also examined the potential effect of cFGF, which is a chimeric molecule based on FGF1 and FGF2 and is said to have a binding ability to FGF receptors 1–4 [53]. Compared to FGF2, cFGF showed an enhanced property to stimulate Tie2 expression maintenance, suggesting its potential usefulness. In this experiment, however, stimulation with cFGF did result in significantly increased activity in either the MEK/ERK or PI3K/Akt pathway. This might be caused by various types of FGF receptors and the FGF family that cFGF can interact with, and thus presents a larger range of stimuli effects, depending on the combinations and receptor [53]. Although the specific signaling pathway that enabled cFGF mediated Tie2 retention could not be discovered in this experiment, we proved that it has a high potential to enhance Tie2 expression. Future work will be necessary to clarify the signal pathway of cFGF in NP cells, as the next step toward cFGF application for supporting the expression of Tie2.

In addition to marking highly proliferative progenitor cells [39], Tie2 is also suggested as a marker to identify ECM-producing cells. Tie2 has specifically been linked to high expression levels of type II collagen [21,29]. Considering their potential application as a regenerative therapy product, we examined the relation between Tie2 expression and type II collagen. Here, we were able to confirm that an increase in TEK expression showed a relationship with COL2A1 expression levels. Moreover, Tie2 positivity rates correlated strongly with type II collagen-positive cells. In summary, in young donors who originally demonstrated Tie2 expression, a high ECM production performance can be obtained by maintaining and enhancing this expression during the culturing process. Additionally, Tie2 positivity rates after culture have been proposed as a marker for type II collagen production performance in the NP cell populations, underlying its potential for future application in regenerative therapeutics. 

## 4. Materials and Methods

### 4.1. Human NP Cell Isolation

Human NP tissues were collected from 20 patients undergoing surgery for lumbar disc herniation at Tokai University Hospital and related facilities (Table 1). All patients provided their informed consent for the collection and use of surgical waste for research purposes. All research procedures described in this study were approved by the Institutional Review Board for Clinical Research, Tokai University (application number 16R-051: 2016/07/15).

NP cells were isolated as previously described [23]. Briefly, the collected surgical NP tissue was washed with excess 0.9% saline and the tissue wet weight was measured. The tissue was cut into pieces 3–5 mm in diameter with sterilized scissors and scalpels. Tissue suspension was thereafter cryopreserved in CELLBANKER (Zenoaq Resource, Fukushima, Japan) until their application in WTC. Alternatively, tissue fragments were further processed for primary cell culture (PCC). For PCC, the shredded NP tissue was carefully transferred to a 50-mm conical tube and centrifuged at 1200 rpm for 5 min at 4 °C. After centrifugation, the supernatant was removed, and the tissue was resuspended in 20 mL of 1:1 TrypLE Express (Thermo Fisher Scientific, Tokyo, Japan) and 10% (*v*/*v*) fetal bovine serum (FBS), minimal essential medium α (MEMα, Fujifilm Wako Pure Chemical Corporation, Osaka, Japan). The suspension was digested with gentle swirling at 37 °C for 1 h. The tissue dissolution state was confirmed and the sample was centrifuged at 1800 rpm for 5 min. Next, the tissue was further digested in a mixture of 15 mL of 10% (*v*/*v*) FBS, αMEM, and 5 mL of 0.25 mg/mL collagenase P (Roche, Basel, Switzerland), which was incubated for 2 h at 37 °C. After digestion, the suspension was again centrifuged, resuspended in 20 mL of 10% (*v*/*v*) FBS MEMα, and filtered through a 40 µm cell strainer (Corning, Corning, NY, USA). The resulting cell suspension was subjected to culture conditions as specified below.

### 4.2. WTC Method

Human NP tissue obtained during surgery was first washed with saline. Tissues were then chopped by using a scalpel blade and surgical scissors to make tissue fragments. The size of the tissue was roughly specified as 3–5 mm in diameter (see Figure 1A). NP tissue fragments were directly applied to a commercially developed NP cell optimized, blended medium (MEMα:32%, DMEM:48%, FBS:20%) provided by our contributing company TUNZ Pharma Co. Ltd. (Osaka, Japan) and cultured on polystyrene 6-well plates (IWAKI, Tokyo, Japan) (Figure 1A). About 0.3 g of NP tissue was seeded per 3 mL culture media in each well (approximately 32 mg/cm^2^). Tissue fragments were cultured at 37 °C in 5% CO_2_ and 5% O_2_ for 14 days without media replenishment. Subsequently, cultured tissue fragments were digested as described above. The derived cells were cultured in an identical fashion as PCC (described below) for another seven days.

### 4.3. PCC Method

Primary NP cells or NP cells isolated following WTC were seeded on standard polystyrene 100-mm plates (Corning) at 37 °C in 5% CO_2_ and 5% O_2_ and cultured in medium as previously specified for seven days without media change. NP cells were seeded at a density of 30,000 cells per plate (545.5 cells/cm^2^). Following seven days in culture, PCC- or WTC-derived cells were applied to their experimental conditions, as indicated below.

### 4.4. Assessment of FGF2 on Tie2 Expression in WTC and PCC

Human NP tissue (*n* = 5, E32, A24, A20, T20, T21, Table 1) was cultured with WTC and PCC methods as described above, with or without supplementation of 10 ng/mL FGF2 (PeproTech, Cranbury, NJ, USA). Cultured cells were retrieved using TrypLE Express for 5 min at 37 °C and subsequently, Tie2 expression rate was determined by flow cytometry. Based on the findings of this study and considerations for potential clinical application and market potential (concerns regarding cost efficacy, etc. [18,43]), we decided to apply expansion culture for the rest of the study as follows: two weeks of WTC without FGF2 stimulation followed by two weeks of monolayer culture (identical to PCC) with 10 ng/mL FGF2 at 37 °C in 5% CO_2_ and 5% O_2_. 

### 4.5. Role PI3K/Akt and MEK/ERK in FGF2 and cFGF Supported Tie2 Maintenance

First, following expansion culture as above described, media were changed to serum-free (in part, to remove any FGF naturally occurring in the serum) and FGF-free media to clearly evaluate the Tie2 maintenance ability of FGF2 and cFGF. At the same time, NP cells were treated with either FGF2 or cFGF (Fujifilm Wako Pure Chemical Corporation, Osaka, Japan) at a concentration of 0, 1, 5, 10, or 20 ng/mL for 24 h. Subsequently, the cells were harvested and analyzed using flow cytometry (*n* = 5, A25, T19, A18, A14, A18-2, Table 1). Concentrations of 10 ng/mL were determined to be optimal and were used for the rest of the study.

Next, in order to assess the effects of FGF2 and cFGF on the ERK and Akt signaling pathways, NP cells were similarly treated with FGF2 and cFGF. To mitigate a potential effect of the FBS in the media, the cells were subjected to identical but serum-free media and cultured for 24 h. The resulting cells were harvested and analyzed by western blot (*n* = 5, A25, A14, A18, A29, T18, Table 1).

Finally, to determine the role of ERK and Akt signaling pathways in supporting the maintenance of Tie2 expression, NP cells following the four weeks of culture were serum-starved for one day, and subsequently subjected for 30 min to (1) 10 ng/mL FGF2 + 20 μM PD98059 (MEK inhibitor, Stemcell Technologies, Vancouver, BC, Canada), (2) 10 ng/mL FGF2 + 1 μM 124015-1MGCN (Akt inhibitor, Merck KGaA, Darmstadt, Germany), (3) 10 ng/mL FGF2, (4) 10 ng/mL FGF2 + 0.1% DSMO, or (5) only serum-starved. Additionally, (6) non-serum-starved cells were also examined. Cultures were subsequently harvested and prepared for western blot (*n* = 2, T18, A20, Table 1) and flow-cytometry analysis (*n* = 5, T18, A18-3, A21, A20-2, A16, Table 1). cFGF was not used in subsequent experiments due to the lack of significant Akt and ERK phosphorylation in the previous experiments and the practical restriction of limited human samples.

### 4.6. Flow Cytometry and Fluorescence-Activated Cell Sorting

NP cells were analyzed using a FACS Calibur flow cytometer (BD Biosciences, Franklin Lakes, NJ, USA) as previously described [23]. Using a propidium iodide-negative gate, only living cells were analyzed. Flow cytometry was used to detect the fraction of NPPC marker Tie2 and disialoganglioside (GD2) [21,23] as well as NP cell marker CD24 [54] expressing NP cells. These NP cells were gated relativity to isotype control antibodies as reported in Sakai et al. [23].

### 4.7. Western Blotting

Intact cells from each 100-mm culture plate were collected and lysed in lysis buffer containing RIPA buffer and 200 mM PMSF. After 60 min on ice, the supernatant was collected. Cells were quantified using the DC protein assay reagent (BIO-RAD, Tokyo, Japan). Protein lysates were resolved by SDS-PAGE (200 V, 22 min) using NuPAGE 4–12% Bis-Tris Gel (Invitrogen), MES Running buffer, and blotted (20 V, 60 min) onto PVDF membranes. Nonspecific signals were blocked with 3% bovine serum albumin. The membranes were incubated with primary antibodies (1:1000) at 4 °C overnight and secondary antibodies (1:2000) at room temperature for 1 h. The primary antibodies used were as follows: Phospho-Erk1/2 (Rabbit mAb #4370, Cell signaling, Danvers, AM, USA), Phospho-Akt (Ser473) (D9E) XP^®^ (Rabbit mAb #4060 Cell signaling), p44/42 Erk1/2 (137F5) (Rabbit mAb #4695 Cell signaling), Akt (pan) (11E7) (Rabbit mAb #4685 Cell signaling), Monoclonal Anti-βActin Clone (AC-15, SIGMA, Missouri, USA). β-actin was used as a loading control. Immunoreactive bands were detected using an Amersham healthcare ECL prime western blotting detection reagent (Cytiva, Tokyo, Japan). The protein signals were quantified by scanning densitometry using a CS analyzer (version 3.0, ATTO, Tokyo, Japan).

### 4.8. Real-Time Polymerase Chain Reaction Analysis

Following four weeks of respective WTC and monolayer cultures, NP cells, supplemented with or without FGF2, were cultured for an additional 24 h with or without a FBS-containing medium. The resulting cell cultures were harvested and prepared for qPCR analysis. Cultured human NP cells (*n* = 1, T16, Table 1) were homogenized using ISOGEN II (Nippon Gene, Tokyo, Japan), and RNA was prepared using an RNAqueous Micro RNA Isolation Kit (Applied Biosystems, Thermo Fisher Scientific, Danvers, MA, USA) following the manufacturer’s instructions. High-Capacity cDNA Reverse Transcription Kits (Applied Biosystems 4387406) were used to reverse transcribe mRNA and to synthesize cDNA. Based on TaqMan gene expression assays (Applied Biosystems), gene expression levels were detected using TaqMan Universal PCR Master Mix and the following primers: TaqMan Gene Expression (human): ACAN (Hs00153936; Applied Biosystems), COL1A2 (Hs01028956; Applied Biosystems), COL2A1 (Hs00264051; Applied Biosystems), and TEK (Hs00919202; Applied Biosystems). PCR was performed using an amplification machine (7500; RealTime PCR Systems) with stage 1 (95 °C for 20 s × 1 cycle) and stage 2 (95 °C for 3 s, 60 °C for 30 s × 50 cycles). C_T_ values were calculated relative to the C_T_ value for the housekeeping gene GAPDH (4333764F; Applied Biosystems). The cut-off value for GAPDH was 0.51.Values were thereafter calculated relative to values obtained from samples that were only serum starved, and were expressed using the 2^−^^ΔΔCT^ method.

### 4.9. Statistical Analysis

All data were processed, analyzed, and illustrated using GraphPad Prism (MacOS v9.0.1(128), GraphPad Software, San Diego, CA, USA, 92108-2711). Pictures and images were formatted using Adobe Illustrator (Adobe, USA). All values are presented as mean (±standard deviation) unless specifically stated otherwise. Statistical differences were determined via one-way or two-way ANOVA, followed by Dunnett’s multiple comparison or Tukey’s comparison test. A *p*-value of <0.05 was considered statistically significant. Significant correlations were identified by the Pearson product-moment correlation coefficient; 0.7 < |r| < 1.0 was considered as a high correlation.

## 5. Conclusions

Collectively, our data combined with previous reporting highlights a connection between FGF2-mediated Tie2 maintenance through the MEK/ERK pathways. Applying WTC culture with FGF2 media supplementation supported the significant enhancement of Tie2 retention in NPPC and thereby offers a promising culture method to support NPPC expansion and experimentation. This Tie2 positivity rate after culture is proposed as a marker of type II collagen production performance in the NP cell populations.

## Figures and Tables

**Table 1:** Details of clinical samples. Age, sex, total weight of IVD tissue collected at the time of surgery, and weight of NP tissue after selection are listed for each sample. LDH: lumber disc hernia, IVD: intervertebral disc, NP: nucleus pulposus.

Code	Age (Year)	Sex	Pathology	IVD Tissue (g)	NP Tissue (g)
E32	32	F	LDH	4.75	4.75
A24	24	M	LDH	3.65	3.30
A20	20	M	LDH	6.98	3.23
T20	20	M	LDH	1.89	1.25
T21	21	F	LDH	2.82	1.81
A25	25	F	LDH	2.07	1.92
T19	19	M	LDH	8.05	7.59
A18	18	M	LDH	5.88	3.73
A14	14	F	LDH	1.74	1.54
A18-2	18	M	LDH	1.74	1.42
A29	29	M	LDH	2.68	1.77
T18	18	M	LDH	3.07	2.70
A18-3	18	M	LDH	2.16	1.26
A21	21	M	LDH	1.75	1.36
A20-2	20	F	LDH	1.06	0.98
A16	16	M	LDH	1.81	1.50
T16	16	M	LDH	2.03	2.03
K30F	30	M	LDH	3.18	1.03
K30M	30	F	LDH	4.26	2.5
K21	21	M	LDH	1.79	0.85

## Data Availability

Data can be requested from the corresponding authors upon reasonable request.

## Supplementary Materials

The following are available online at https://www.mdpi.com/article/10.3390/ijms22094723/s1, Figure S1: Assessment of different concentrations of 124015-1MGCN on cell viability and Tie2 expression maintenance.
