# Supplementary material for: Effect of Whole Tissue Culture and Basic Fibroblast Growth Factor on Maintenance of Tie2 Molecule Expression in Human Nucleus Pulposus Cells
Source: Int J Mol Sci. 2021 Apr 29;22(9):4723. doi: 10.3390/ijms22094723 (PMC8124367; doi:10.3390/ijms22094723)
Supplement: Supplementary file 1 [file ijms-22-04723-s001.zip › ijms-1186726-supplementary.pdf]

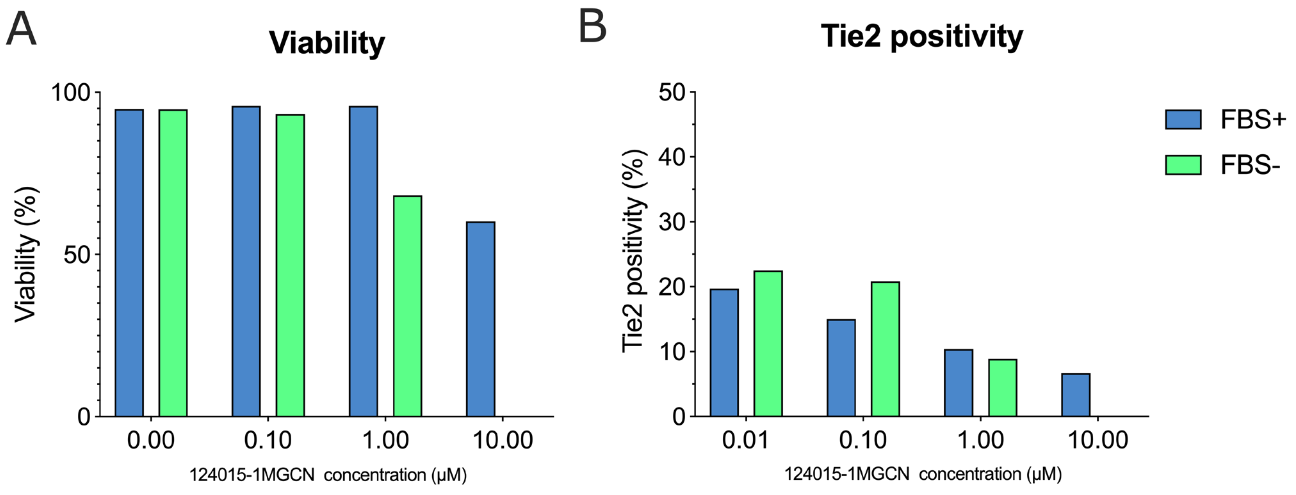

SUPPLEMENTARY FIGURE 1 - Assessment of different concentrations of 124015-1MGCN on cell viability and Tie2 expression maintenance. Human nucleus pulposus cells (n=1, T23) seeded at 50,000 cells/well on 6-well plate. Overnight, the cells were serum starved (FBS-, green) or maintained in serum (fetal bovine serum: FBS+, blue) and concurrently treated with Akt inhibitor 124015-1MGCN at concentrations ranging from 0 – 10 μM. Subsequently, using flow cytometry analysis, the cells were assessed on (A) viability through propidium iodide staining or (B) on Tie2 expressing. The results revealed that cell viability could be fully maintained up to 1μM when supported in FBS containing media, or at 0.1μM in serum starved conditions. Tie2 positivity showed a concentration dependent decrease for both serum starved and serum containing conditions
